# Accuracy of visual and radiographic ICDAS-merged criteria for secondary caries on permanent posterior teeth

**DOI:** 10.4317/jced.63565

**Published:** 2026-04-25

**Authors:** Yaysa Vásquez, Margarita Usuga-Vacca, Stefania Martignon, Andrea Cortes

**Affiliations:** 1 UNICA – Caries Research Unit, Research Department, Universidad El Bosque

## Abstract

**Background:**

This in vitro study evaluated the diagnostic accuracy of visual and radiographic assessments for secondary caries (ICDAS-merged) on posterior permanent teeth.

**Material and Methods:**

Teeth were obtained from a university tooth bank and selected based on the presence of occlusal or proximal restorations. Two trained examiners assessed 296 restored surfaces using ICDAS-merged secondary caries criteria, grouping scores into sound (0), initial (1-2), moderate (3-4), and extensive (5-6). Scores were validated against a histological gold standard using three diagnostic thresholds: (1) sound vs. initial+moderate+extensive; (2) sound+initial vs. moderate+extensive; and (3) sound+initial+moderate vs. extensive. Analyses included Spearman correlation, weighted kappa, and calculation of sensitivity, specificity, and accuracy.

**Results:**

After sample evaluation included 129 premolar surfaces and 167 molar surfaces, restored either with amalgam (n = 194) or composite resin (n = 102). Both methods showed substantial inter- and intra-examiner reproducibility (kappa: 0.70-0.80). Correlation with histology was substantial for both visual (=0.73) and radiographic (=0.70) assessments. At the threshold of sound vs. initial+moderate+extensive, specificity was high (visual: 0.85; radiographic: 0.93), while sensitivity was higher for visual (0.80) than for radiographic criteria (0.68). Overall accuracy was high for both methods (visual: 0.83; radiographic: 0.81). Across restorative materials, visual assessment showed consistent accuracy for amalgam and composite restorations. In contrast, radiographic assessment showed lower sensitivity for amalgam (0.61) and composite (0.64) restorations at the same threshold. ICDAS-merged visual criteria demonstrated strong accuracy and reproducibility for detecting secondary caries in restored permanent teeth, regardless of restorative material.

**Conclusions:**

Radiographic criteria exhibited high specificity but lower sensitivity, particularly for early lesions, highlighting the value of visual inspection as a reliable method for detecting secondary caries in clinical settings.

## Introduction

Secondary caries is a carious lesion that develops adjacent to a restoration (1) because of an imbalance between mineral loss and gain at the dental biofilm-substrate interface (1 , 2). It is one of the most common conditions in adult patients at a high risk of caries, occurring mainly adjacent to occlusal restorations and at the gingival margins of a restoration (3). Secondary carious lesions have been conventionally detected using systems that mainly assess the condition of restorations and determine the presence/absence and extent of dental caries as an additional criterion to determine the need for permanence or replacement of the restoration in the mouth, under observation (4 , 5). Failure to recognise secondary caries results in high levels of restoration replacement as well as subsequent weakening of the remaining tooth structure and shortening of its life cycle (6 , 7) . The classification system for secondary caries proposed by the International Caries Detection and Assesment System (ICDAS) in (ICCMS): Caries Associated with Restoration/Sealants (CARS) has four categories: sound 'S' (code 0 = no caries adjacent to restoration/sealant), initial 'I' (code 1 = first visible change in enamel adjacent to r/s after drying and code 2 = visible change adjacent to r/s, no cavitation), moderate 'M' (code 3 = loss of enamel continuity adjacent to r/s, cavity less than 0.5 mm and code 4 = caries in enamel/dentine/cement adjacent to r/s with underlying dark shading of dentine), and extensive 'E' (code 5 = visible cavity 0.5 mm and code 6 = cavity with visible dentine in floor and walls adjacent to r/s). This classification system is applicable to visual and radiographic findings (8). For the purposes of this study, the term "ICDAS-merged criteria for secondary caries" will be used, in alignment with the 2020 Consensus on Terminology in Dental Caries and Caries Management (1). The implementation of this classification system needs to be determine by comparing its operational characteristics with those of a gold standard (9). Previous studies have compared the performance of the ICDAS visual criteria for secondary caries with that of the histological assessment (10 - 12). Two of these studies conducted visual and radiographic assessments using the removal of restorations in resin-treated primary molars and in amalgam class II restorations as the histological standard. In these cases, both methods exhibited similar accuracy values, although radiographic assessment was less accurate for lesions located in dentin. Meanwhile, in permanent molars with Class II amalgam restorations, the visual assessment proved to be more sensitive (10 , 11). Although these studies have investigated the operational characteristics of the ICDAS visual and radiographic criteria for secondary caries, significant differences were observed between them in terms of reference histology, specimen, restorative material and surface. These studies have evaluated the visual and radiographic ICDAS criteria for secondary caries, focused exclusively on primary teeth or on a single type of restorative material, such as amalgam or resin. However, in clinical practice, permanent teeth restored with both materials are common and present distinct diagnostic challenges due to their optical and radiographic properties. Therefore, it is essential to determine the accuracy of the ICDAS-merged criteria for secondary caries in permanent teeth restored with amalgam and resin to ensure accurate and evidence-based clinical decision-making. This study aimed to evaluate the sensitivity, specificity and accuracy of the ICDAS-merged visual and radiographic criteria for secondary caries in natural teeth, using the ICDAS histological criteria as a reference.

## Material and Methods

**Figure 1:**
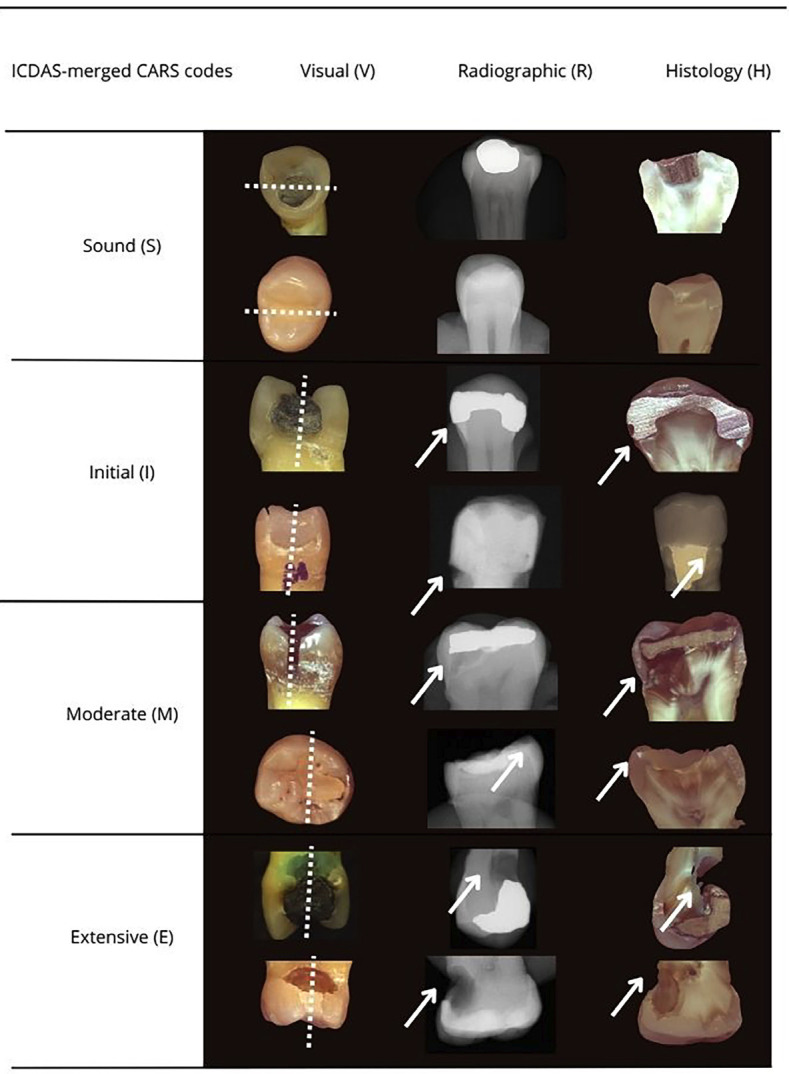
Visual (V) and Radiographic (R) ICDAS-merged classification of secondary carious lesions before sectioning extracted teeth and histological grouping after sectioning.

This in vitro study was approved by the Research Ethics Committee of the Universidad El Bosque - UEB (code NUR-2021-187) and complied with the recommendations for the design of precision studies for diagnostic tests Standards for Reporting of Diagnostic Accuracy (STARD) (13 , 14). Three ICDAS criteria experts, calibrated to the secondary caries criteria, determined the agreement of the visual and radiographic diagnoses with the ICDAS-merged criteria for secondary caries, grouping the ICDAS severities into sound (0), initial (ICDAS 1 and 2), moderate (ICDAS 3 and 4) and extensive (ICDAS 5 and 6) (8). A sample size of 30 areas per ICDAS-merged for secondary caries severity category was calculated using the Lachin formula (1981) (15). Based on the validation studies of ICDAS II (9), and studies of the prevalence of secondary caries (16), P &lt; 0,05 and 0,80 power. This study included permanent premolars and molars with occlusal or occlusal/proximal amalgam or resin restorations that had been extracted. Teeth were obtained from the UNICA-Caries Research Unit, Universidad El Bosque tooth bank. Two examination surfaces per sample were selected by an external calibrated examiner, when the dimensions of the sample allowed for more than one section to be obtained, or when two opposing decay surfaces were present within the same section. Then, Visual, radiographic and histological assessments of the surfaces were conducted by two examiners, followed by re-evaluations after 1 week. ICDAS-merged visual assessment (V) criteria for secondary caries The area of interest was marked on each. Two of the authors (MU and AC) assessed the visual tactile appearance of the selected area on the tooth, using the wit a blunt-tipped probe (OMS), according to the ICDAS-merged codes for secondary caries as sound (S) or with initial (I), moderate (M) or extensive (E) secondary carious lesions (Fig. 1).

1

All lesions were assessed twice with 1 week in between the two recordings. In case of disagreement between examiners, consensus was achieved. ICDAS-merged radiographic assessment criteria for secondary caries Digital standardised radiographs (R) were taken using a radiovisiograph (Xios Supreme Dentsply Sirona, Germany) and a portable X-ray unit (DEcowin DX 3000) with the following configurations: exposure time of 0.08 s and 23 cm between the opening of the X-ray head and the sample. The radiographs were evaluated and classified using the following ICDAS-merged radiographic codes for secondary caries: no radiolucency (S); initial radiolucency in the enamel (I); radiolucency involving the middle third of the dentine-moderate (M); and radiolucency involving the inner third of the dentine-extensive (E) (Fig. 1). All lesions were assessed twice with 1 week in between the two recordings. In case of disagreement between examiners, consensus was achieved. - Histological assessment ICDAS-merged criteria Each tooth was hemi-sectioned perpendicular to the evaluated surface using a microtome (Buehler, IsoMet 1000) with a diamond disc (Buehler, four in Diam × 0.012 in at 350 rpm). Images of the two histological sections were taken using a stereomicroscope (8×) (Olympus SZ- 61TRLED, Japan). Hemi-sections were evaluated and classified using the ICDAS histological criteria proposed by Ekstrand et al. (1997) (17 , 18). The demineralisation depth was evaluated using the following ICDAS-merged codes: S, no demineralisation; I, demineralisation in the enamel 1 and 2 codes; M, demineralisation to the outer and middle third of the dentin 3 and 4 codes; and E, demineralisation to the inner third of the dentin, 5 and 6 codes (8). Disagreements were resolved by discussion. The two examiners established a diagnostic criterion. All lesions were assessed twice with 1 week in between the two recordings. In case of disagreement between examiners, consensus was achieved. - Statistical analysis The inter- and intra-examiner reproducibility of the ICDAS-merged visual and radiographic criteria for secondary caries were determined using the weighted kappa statistic. To obtain the accuracy of the ICDAS-merged visual and radiographic criteria for secondary caries with those of histology, the following thresholds were established: sound vs. initial + moderate + extensive; sound + initial vs. moderate + extensive; and sound + initial + moderate vs. extensive. These thresholds were selected to determine the ability of the tests to detect sound, initial and extensive lesions (18). The operational characteristics, namely, specificity, sensitivity and accuracy, of the ICDAS-merged visual and radiographic criteria for secondary caries, were compared with those of the histological criteria at a 5% significance level. The entire analytical workflow was implemented in R version 4.5.1. The results were interpreted globally using the scale proposed by Landis and Koch (1977) (19 , 20) differentiating between restorative materials (amalgam or resin).

## Results

This study included 296 posterior tooth surfaces with amalgam (n = 194) or resin (n = 102) restorations histologically classified as healthy (n = 184) or with initial (n = 54), moderate (n = 31) and extensive (n = 27) carious lesions, as depicted in Figure 1. The intra- and inter-examiner reproducibility exhibited substantial agreement using visual and radiographic detection systems (Table 1).

Table 1

Spearman correlation vs histology was substantial for the visual and radiographic criteria (p=0.73, p=0.70) (Table 2).

Table 2

Accuracy of ICDAS-merged visual and ICDAS-merged radiographic criteria for secondary caries, versus histology The operational characteristics of the visual criteria showed an almost-perfect specificity in all thresholds (0.85-0.98) compared with those of the radiographic criteria (0.92-0.98). The visual criteria exhibited a higher sensitivity (almost perfect) with the threshold sound vs. initial + moderate + extensive (0.80) than the radiographic criteria with the same threshold (0.62) (Table 3).

Table 3

When the performances of the methods were compared between the dental materials, no significant differences were observed, with a higher sensitivity for the amalgam (0.82) in visual exams for initial caries detection, and the highest sensitivity values in composite on radiographic detection at the same threshold (0.64) (Table 4).

Table 4

The accuracy of both criteria ranged from being substantial to almost perfect. Meanwhile, the accuracy of the ICDAS-merged visual criteria for secondary caries ranged from being substantial to almost perfect, the ICDAS-merged radiographic criteria for secondary caries demonstrated substantial accuracy but low sensitivity in distinguishing carious surfaces from healthy ones and in detecting initial lesions (Tables 3,4).

## Discussion

This study compared the ICDAS-merged visual and radiographic criteria for secondary caries with the histological ICDAS criteria, which have sufficient evidence as a useful reference for the study of coronal caries (21) and secondary caries (11 , 12). The diagnosis of secondary caries beneath restorations involves two sequential clinical decisions: identifying the presence of a lesion and determining its extent to guide appropriate management. This study aimed to evaluate the accuracy for secondary caries of visual inspection and intraoral radiography (ICDAS-merged) on the same set of restored surfaces, using histological examination as the reference standard. To enhance clinical applicability, the diagnostic scale was condensed into three operational thresholds aligned with decision-making scenarios: S vs I+M+E (presence of any lesion), S+I vs M+E (intervention threshold), and S+I+M vs E (extensive lesion). Herein, the ICDAS criteria were used as the histological reference standard, following the method described by Diniz et al. (2016) (11). However, unlike the approach, this study included both resin-based composites and amalgam restorations. No restorative material was removed during the histological assessment to prevent inadvertent loss of the study surface, which could potentially influence the favourable results obtained. The agreement between the ICDAS-merged visual criteria for secondary caries and histology criteria, was almost perfect for cavitated carious lesions (moderate and extensive). However, for initial lesions, the agreement was substantial, probably due to several factors, such as less mineral loss and greater difficulty in detecting the presence of a restorative material, as amalgam and resin can cause marginal discoloration of the tooth structure, unrelated to caries, which affects the optical properties of the surface (22 - 24). When detecting with radiograph initial carious lesions, the sensitivity was lower (0.62-0.81). This suggests that the method is less effective in identifying early-stage cases, potentially limiting its utility in preventive interventions. Furthermore, these findings align with those reported in a previous study by Diniz et al., (2016b) (12), reinforcing the validity of the current results. However, it is worth noting that the specificity reported in the current study was higher than in Diniz et al.'s findings Diniz et al., (2016) (12). The material-specific pattern mirrors the trends observed in the overall analysis: visual examination tends to yield higher sensitivity, particularly for early-stage disease, whereas radiographic assessment maintains superior specificity, which is critical for confirming and delineating lesion extent. Differences between amalgam and composite restorations were small to moderate and, aside from the influence of wider confidence intervals observed in composite surfaces, do not alter the clinical conclusions. For both materials, the diagnostic threshold of S+I+M vs. E demonstrated excellent accuracy (Acc= 0.96-1.00). Accordingly, the findings support a complementary strategy in clinical practice: visual inspection as the first-line approach, with radiographic imaging used to corroborate and characterize lesions, regardless of the restorative material. The visual assessment sensitivity values (0.80-0.82) obtained for amalgam-retained molar were higher than those reported by Diniz et al. (2016b) which was 0.20 (12). These differences could be associated with the histological reference standard used; while this approach preserved the integrity of the study surfaces, it may have influenced the sensitivity values, particularly for early lesions. Future studies should consider alternative methods to mitigate verification bias while maintaining sample integrity. Additionally, with the Minamata Convention, action has been taken to progressively reduce the use of amalgam as a dental restorative material worldwide; therefore, research on secondary caries detection systems should aim to improve their performance in detecting lesions in teeth restored with mercury-free materials (25 , 26). Despite the limitations in sensitivity for the early detection of carious lesions, the specificity values achieved by ICDAS-merged confirmed its substantial to almost-perfect accuracy for secondary caries validation. The intra- and inter-examiner reproducibilities achieved were good to almost perfect, indicating examiner reliability in the use of the ICDAS-merged criteria (27). This study provides valuable evidence for the clinical and research use of the ICDAS-merged visual and radiographic criteria for secondary caries.

## Conclusions

This study validated the ICDAS-merged visual and radiographic criteria for detecting secondary caries on occlusal and proximal surfaces of restored teeth. Both methods demonstrated substantial correlation with the histological gold standard (r = 0.70 and 0.73, respectively), with visual assessment showing higher sensitivity. While both visual and radiographic methods exhibited high specificity, visual inspection proved to be more reliable for detecting secondary caries, particularly in early and moderate stages. The findings support a complementary diagnostic strategy in which visual inspection serves as the primary modality for initial lesion detection, and radiographic evaluation is employed to confirm lesion extent when clinically indicated. This approach is consistent with evidence-based decision-making and enhances diagnostic accuracy in the assessment of secondary caries beneath restorations. Further in vitro studies are warranted to differentiate between permanent and primary teeth, as well as ex vivo investigations to detect, assess severity, and consider lesion activity using the ICDAS-merged criteria for secondary caries.

## Figures and Tables

**Table 1:** Reproducibility of examiners for visual and radiographic assessment with ICDAS-merged criteria for secondary caries.

Examiner	Visual ICDAS-merged		Radiographic ICDAS-merged	
	Inter-Examiner	Intra-Examiner	Inter-Examiner	Intra-Examiner
	K	K	K	K
1	0.72	0.75	0.76	0.73
2	0.75	0.80	0.70	0.71

Weighted Kappa (WK)

**Table 2:** Cross-tabulation of Visual and Radiographic ICDAS-merged criteria for secondary caries (n=296).

Histology ICDA	Visual ICDAS-merged				
	Sound	I	M	E	Total
Sound	157	19	1	2	179
I	16	23	5	1	45
M	11	10	24	1	46
E		2	1	23	26
Total	184	54	31	27	296
Spearman Correlation 0.73					
Histology ICDAS	Radiographic ICDAS-merged				
	Sound	I	M	E	Total
Sound	172	35	6	1	214
I	5	9	5		19
M	7	9	18	4	38
E		1	2	22	25
Total	184	54	31	27	296

Spearman Correlation 0.70

**Table 3:** Operative characteristics of Visual and Radiographic ICDAS-merged criteria for secondary caries.

Tresholds	Visual ICDAS-merged for Secondary Caries					
	Se	CI 95%	Sp	CI 95%	Acc	CI 95%
S vs. I+M+E	0.80	(0.71-0.87)	0.85	(0.79-0.90)	0.83	(0.78-0.87)
S+I vs. M+E	0.84	(0.72-0.92)	0.90	(0.85-0.93)	0.89	(0.85-0.92)
S+I+M vs. E	0,85	(0.66-0.95)	0.98	(0.96-0.99)	0.97	(0.95-0.99)
Treshholds	Radiographic ICDAS-merged for Secondary Caries					
	Se	CI 95%	Sp	CI 95%	Acc	CI 95%
S vs. I+M+E	0,62	(0.52-0.71)	0.93	(0.88-0.96)	0.81	(0.76-0.86)
S+I vs. M+E	0.79	(0.66-0.88)	0.92	(0.88-0.95)	0.90	(0.86-0.93)
S+I+M vs. E	0.81	(0.61-0.93)	0.98	(0.96-0.99)	0.97	(0.94-0.98)

p< 0.05. Sensitivity (Se), Specificity (Sp) and Accuracy (Acc)

**Table 4:** Operative Characteristics of Visual and Radiographic ICDAS-merged criteria for secondary caries according to dental material.

Material	Visual ICDAS-merged						
	Thresholds	Se	95% CI	Sp	95% CI	Acc	95% CI
Amalgam(n=194)	S vs. I+M+E	0.82	(0.72-0.90)	0.82	(0.72-0.90)	0.82	(0.76-0.87)
	S+I vs. M+E	0.82	(0.67-0.92)	0.87	(0.67-0.92)	0.86	(0.81-0.91)
	S+I+M vs. E	0.80	(0.56-0.94)	0.99	(0.56-0.94)	0.96	(0.92-0.98)
Composite(n=102)	S vs. I+M+E	0.74	(0.54-0.88)	0.90	(0.80-0.77)	0.84	(0.75-0.91)
	S+I vs. M+E	0.88	(0.63-0.98)	0.94	(0.85-0.98)	0.93	(0.85-0.97)
	S+I+M vs. E	0.99	(0.59-0.99)	0.99	(0.95-0.99)	0.99	(0.95-0.99)
Material	Radiographic ICDAS-merged						
	Thresholds	Se	95% CI	Sp	95% CI	Acc	95% CI
Amalgam(n=194)	S vs. I+M+E	0.61	(0.50-0.72)	0.92	(0.85-0.96)	0.79	(0.73-0.84)
	S+I vs. M+E	0.73	(0.57-0.85)	0.90	(0.85-0.94)	0.87	(0.81-0.91)
	S+I+M vs. E	0.75	(0.50-0.91)	0.98	(0.95-0.99)	0.96	(0.92-0.98)
Composite(n=102)	S vs. I+M+E	0.64	(0.45-0.80)	0.94	(0.84-0.98)	0.83	(0.74-0.90)
	S+I vs. M+E	0.94	(0.71-0.99)	0.95	(0.87-0.99)	0.95	(0.88-0.98)
	S+I+M vs. E	0.99	(0.59-0.99)	0.98	(0.93-0.99)	0.98	(0.93-0.99)

p< 0.05. Sensitivity (Se), Specificity (Sp) and Accuracy (Acc)
